# Quantitative analysis of therapeutic response in psoriatic arthritis of digital joints with Dual-energy CT iodine maps

**DOI:** 10.1038/s41598-020-58235-9

**Published:** 2020-01-27

**Authors:** Reina Kayama, Takeshi Fukuda, Sho Ogiwara, Mami Momose, Tadashi Tokashiki, Yoshinori Umezawa, Akihiko Asahina, Kunihiko Fukuda

**Affiliations:** 1 Department of Radiology, The Jikei University School of Medicine, Tokyo, Japan; 2 Department of Dermatology, The Jikei University School of Medicine, Tokyo, Japan

**Keywords:** Tendons, Tendons

## Abstract

The aim of this study was to investigate the feasibility of quantitative assessment of the therapeutic response in psoriatic arthritis (PsA) by measuring iodine uptake using a Dual-energy CT (DECT) iodine map. The study included 74 symptomatic and 74 matching non-symptomatic joints of 26 consecutive PsA patients who underwent two contrast enhanced DECTs of the hand or foot, pre and post medical interventions. Symptomatic and matched non-symptomatic control joints were scored with the PsA DECT Scoring System (PsADECTS), which was derived by modifying the PsA MRI Scoring System (PsAMRIS), a recently validated scoring system that assesses PsA changes on MRI. Quantified iodine uptake measured using the DECT iodine map was compared to the PsADECTS score. Efficacy of PsA treatment was confirmed by the improved clinical findings. Both PsADECTS and iodine uptake also showed significant improvement after treatment (Wilcoxon signed-rank test: z = 7.38, p < 0.005; z = 6.20, p < 0.005, respectively). The treatment effects of PsADECTS score and iodine uptake showed a good correlation with each other (Spearman’s ρ = 0.58 p < 0.005). Inter-reader agreement for PsADECTS score and iodine uptake were either moderate or good. In conclusion, our study showed that the DECT iodine map is a valid tool for quantitative assessment of the therapeutic response of PsA.

## Introduction

Psoriatic arthritis (PsA) is a chronic immune-mediated inflammatory arthritis, which is observed in 6–42% and 10.5–14.3% of psoriasis patients in western countries and in Japan, respectively^1–3^. Conventionally, objective imaging modalities such as X-ray images were used to monitor the therapeutic effect of active PsA by assessing bone erosion and joint destruction. The European League Against Rheumatism (EULAR) recommended that patients with arthritis should be referred to a rheumatologist within 6 weeks after the onset of symptoms to improve outcome^4^. Moreover, with the advanced therapeutic management of PsA, such as the introduction of biologic agents, which have excellent therapeutic effects on enthesitis and synovitis, imaging modalities that can evaluate inflammation, before bony destruction become apparent, are required^5^.

Magnetic resonance imaging (MRI) and ultrasound (US) are the modalities of choice to evaluate the severity of inflammation and are now widely used in daily practice^6–9^. The PsA MRI Scoring System (PsAMRIS) was developed in 2009, by the Outcome Measures in Rheumatology Clinical Trials (OMERACT), in an effort to obtain a semiquantitative assessment of PsA changes^10^. Since its development, several studies have shown the feasibility of MRI and US investigation, which aided in selecting further management according to the imaging findings^7,11–13^. Nonetheless, MRI holds several disadvantages, especially when evaluating digital joints. Firstly, the peripheral joints are more prone to producing artifacts, which become worse in the distal interphalangeal (DIP) joints, which are commonly affected in PsA. Insufficient fat saturation is also encountered, which may lead to misinterpretation of the severity of inflammation. Secondly, the spatial resolution of MRI is inadequate. The usual MRI slice thickness is 3 mm with the spin-echo technique, which is too thick to evaluate the small digital joints. Thirdly, although gapless thin-section MR images can be obtained to create three orthogonal reconstructed planes of each digit with a gradient-echo technique, image quality is not always sufficient. Fourthly, MRI commonly has longer waiting lists and scanning time, therefore it is more difficult to maintain the designated position of the hand or foot, compared to CT. US also has its disadvantages in the evaluation of digital PsA. Restricted access makes it impossible to evaluate certain anatomic sites, such as the interosseous lateral aspects of metacarpophalangeal (MCP) joints. Reproducibility of images with US is also limited by the fact that it is an examiner-dependent modality^8^. Computed tomography (CT) has the potential to overcome these problems with a much shorter scanning time, but it has been used mainly for evaluating structural changes and not inflammatory lesions^14^. In conventional single-energy CT, even with a contrast medium, images were not sufficient to delineate inflammatory lesions of PsA. Introduction of DECT iodine map enabled the emphasis of iodine medium to increase contrast resolution.

Dual-energy CT (DECT) iodine mapping was first introduced into the clinical setting in 2005, which enabled quantitative assessment of iodine contrast enhancement and the creation of iodine maps, by acquiring two different sets of data from two different energy levels of X-rays^15^. DECT iodine mapping is highly sensitive in depicting inflammation around the joints in PsA^16^ and can be used for semiquantitative assessment by using a modified version of PsAMRIS^17^, a recently validated method that assesses PsA changes on MRI^12,13^. In this study we have defined this scoring system as the PsA DECT Scoring System (PsADECTS), which is shown to be as useful or may be superior as the MRI in assessing inflammation of small joints^17^.

However, all scoring systems using MRI, US and DECT are semiquantitative, which relies on subjective assessment by clinicians. Furthermore, applying these scoring systems to each digital joint can be a time-consuming procedure, which may not be suitable in daily practice. The DECT iodine map enables iodine quantification with automatic calculation through drawing the Region of Interest (ROI), allowing a more objective method to measure the activity of inflammation through a quantitative approach. Therefore, the aim of this study was to assess the validity of quantitative assessment of the therapeutic response of PsA with DECT iodine map by comparison with the PsADECTS.

## Materials and Methods

### Patients

This retrospective study included consecutive patients who were diagnosed with symptomatic PsA of the digital joints of the hand and foot at the dermatology outpatient clinic, who underwent two contrast enhanced DECT scans pre and post medical interventions from December 2014 to April 2017.

Symptomatic joints were chosen according to patient presentation. As the control, matching non-symptomatic joints were chosen by using the following criteria; criteria 1: non-symptomatic joint on the same digit (If more than 1 joint was present on the same digit, then the most distal joint was chosen. This is due to the difficulty in delineating the boundaries of adjacent MCP joints when placing the ROI). In the absence of an appropriate joint, criteria 2: adjacent joint of the closest digit (excluding the first digit, due to lack of equivalent proximal interphalangeal (PIP) and DIP joints) was applied. As with criteria 1, if more than 1 joint was present on the adjacent digit, then the most distal joint was chosen. If no non-symptomatic joint matched criteria 1 or 2, criteria 3: any closest non-symptomatic joint (excluding the first digit) was selected. If more than 1 joint fit the criteria, then a joint was chosen at random. When placing the ROI was difficult in small non-symptomatic joints, a larger adjacent joint was used.

For clinical assessment, the Classification Criteria for Psoriatic Arthritis (CASPAR) and Psoriasis Area Severity Index (PASI) scores were calculated prior to treatment, and Visual Analog Scale (VAS) was measured at the time of scanning. Blood sample data closest to the time of the scan was also collected as a clinical marker. The mean and standard deviation of interval between the time of blood test and scanning was calculated. For C-reactive protein (CRP), values of “less than 0.04 mg/dl” were handled as “0.04 mg/dl” in this analysis for descriptive purposes. Neutrophil-lymphocyte ratio (NLR) and platelet-lymphocyte ratio (PLR) were also collected as they are good indicators of systemic inflammation and were also shown to have positive association with PASI scores^18^.

Institutional review board approval was obtained (approval number: 29–124(8740) The Jikei University School of Medicine). All experiments were performed in accordance with hospital guidelines and regulations. Informed consent was obtained from all patients for each DECT examination.

### DECT protocol

Affected hand or foot of all patients were scanned with the SOMATOM Definition Flash (Siemens Healthineers) in DE mode with the following parameters: tube voltage of 80 kV and 140 kV with the use of a 0.4 mm tin filter, 250 and 125 effective mAs (11.60mGy), 0.5-second rotation time, 40 × 0.6 mm collimation, and pitch of 0.6. The contrast medium, iohexol 100 mL was injected at a rate of 1.5 mL/sec. Scanning was started at 120 seconds after injection.

### DECT image processing

**Figure 1:**
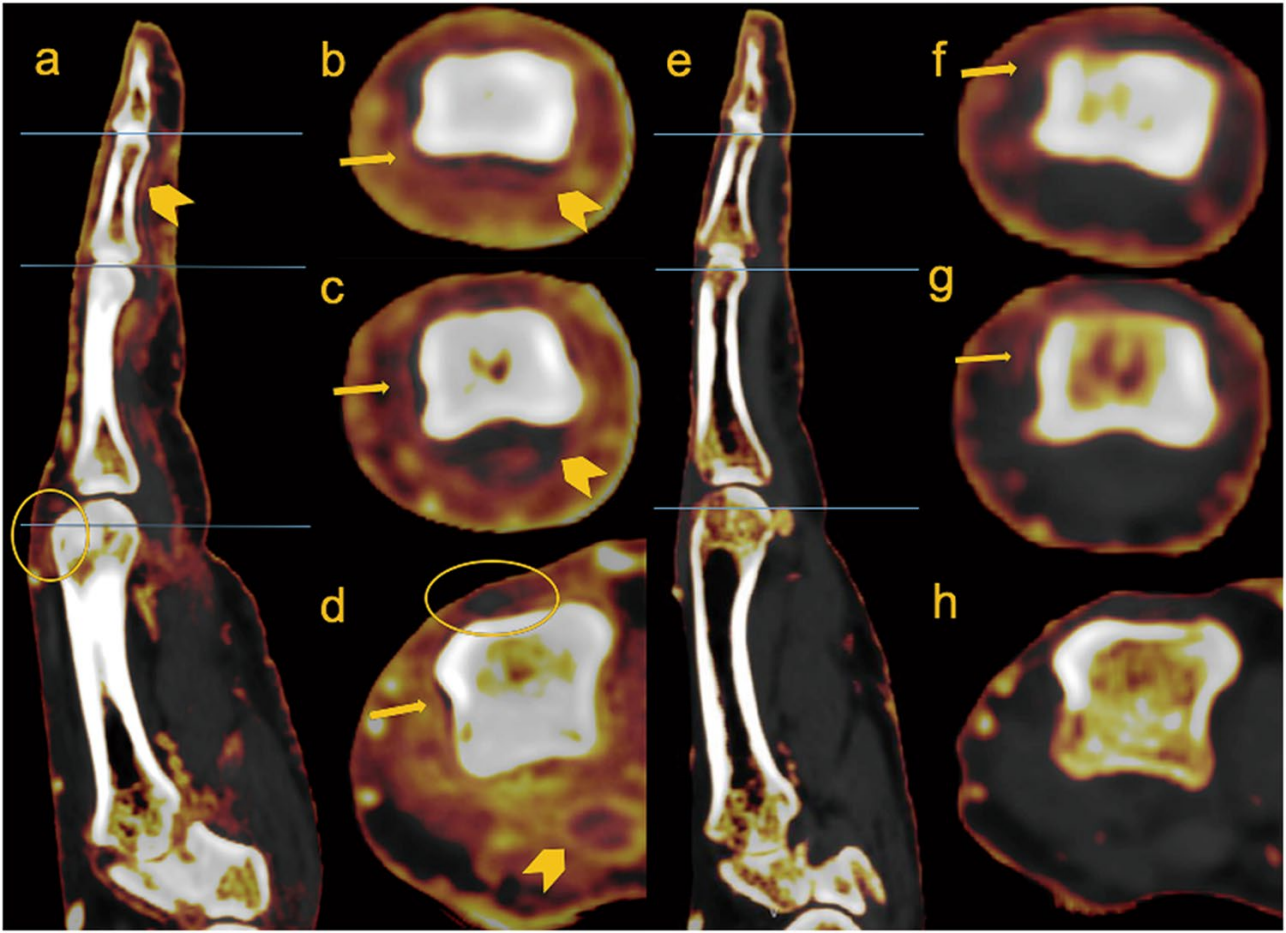
Sagittal and axial images of the right 2^nd^ digit, pre (**a**–**d**) and 1year post treatment (**e**–**h**) with Adalimumab, of 80-year-old male PsA patient. Circular enhancement along joint capsule suggests synovitis (arrows in **b**–**d**), flexor tenosynovitis (arrow heads in **a**–**d**) and obvious periarticular enhancement is seen on the before treatment. Extensor peritendonitis (circle in a) is also seen on the MCP, pre-treatment. Those findings improved on the post-treatment with only a mild synovitis remaining (arrows in **f**,**g**).

All images were reconstructed from DE data with 1.0 mm slices in 0.7 mm increments. A commercial workstation (Syngo Dual-energy, Liver VNC; Siemens Healthineers) was used for three-material decomposition analysis. To determine the composition of three materials; for example, non-fat soft tissue, fat and iodine; three-material decomposition can be performed by first computing the mass fraction of two materials using conventional two-material DECT techniques and attributing the missing fraction to the third material. A three-material decomposition algorithm provides information of attenuation and iodine concentration, which can be used to create iodine map image. The iodine map image was created in three orthogonal planes, with axial, coronal and sagittal planes for each joint (from first to fifth digit)^16^. Window center and window width of images were fixed as 55 and 70, respectively, for image standardization (Fig. 1).

### PsA DECT iodine map scoring system (PsADECTS)

All images were evaluated by two qualified radiologists (R.K. and T.F., with 4 and 8 years of experience in interpreting musculoskeletal CT images, respectively) independently, while blinded to clinical data and other images. The symptomatic and non-symptomatic joints were scored according to PsADECTS^17^. PsADECTS was scored out of 10: synovitis, flexor tenosynovitis, extensor peritendonitis (each scored out of 3) and periarticular inflammation (scored 1 if present). Grades for synovitis was defined as follows: no contrast enhancement was scored as grade 0; enhancement of less than the semicircle of the joint capsule was scored as grade 1; enhancement of half or more of the semicircle but less than the entire circle of the joint capsule was scored as grade 2; and enhancement of the entire circumference of the joint capsule was scored as grade 3. Flexor tenosynovitis and extensor peritendonitis was defined as contrast enhancement around the tendons. If the maximum thickness of enhancement was less than half the tendon thickness, it was scored as grade 1. Enhancement of half to less than full tendon thickness was scored as grade 2, and enhancement of the full tendon thickness or more was scored as grade 3 (Table 1).

**Table 1:** PsA DECT iodine map scoring system (PsADECTS).

Grade (degree of enhancement)	0	1	2	3
Synovitis	No enhancement	Less than semicircle of the synovium capsule	Half or more than the semicircle but less than the entire circle of the capsule	Entire circumference of the capsule
Flexor tenosynovitis	No enhancement	Less than half of tendon thickness	More than half but less than full tendon thickness	Full tendon thickness
Extensor peritendonitis	No enhancement	Less than half of tendon thickness	More than half but less than full tendon thickness	Full tendon thickness
Periarticular inflammation	No enhancement	Periarticular enhancement	N/A	N/A

If the readers disagreed, a subsequent discussion was held to produce an adjusted consensus score.

Joints of the first to the fifth MCP, the second to the fifth PIP, the second to the fifth DIP and the first interphalangeal (IP) joints were evaluated.

### Iodine uptake measurement

**Figure 2:**
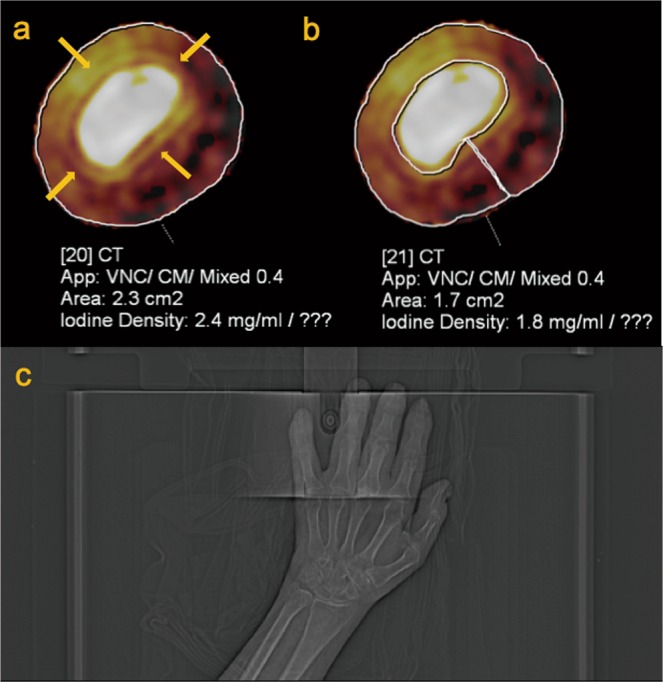
Axial image of the left 5^th^ DIP joint (**a**,**b**) and a scout image left hand (**c**) of 42-year-old male PsA patient. Circular enhancement along joint capsule suggests synovitis (arrow) and obvious periarticular enhancement is also seen. Calculated iodine concentration (which is shown as Iodine Density in figures) is different when ROI included the bone (**a**) and when the bone is excluded (**b**).

To measure the amount of iodine, ROI were placed free-handedly by the assessing radiologist, at the level where the joint was most affected according to PsADECTS on the axial plane of the finger. As shown on the Fig. 2, when the ROI is placed on the DECT iodine map, iodine uptake area and the mean attenuation is displayed. The direct comparison of calculated iodine concentration may lead to misinterpretation of the therapeutic effect in PsA. This is because swelling due to edema is likely to reduce the iodine concentration while increasing the unenhanced background proportion regardless of active inflammation, and normal vasculature in a non-swollen finger may contribute to an increase in iodine concentration in less severe cases. Therefore, we calculated the estimated iodine volume (calculated by iodine concentration × pixel numbers) when determining therapeutic effect. Furthermore, ROI was drawn excluding the bone, because the bone affects iodine calculation due to its high X-ray attenuation (Fig. 2b).

### Statistical analysis

The Wilcoxon rank sum test and Fisher’s exact test were used to evaluate differences in patient characteristic between men and women.

The treatment effect of PsADECTS score and iodine uptake in symptomatic joints were subtracted by the matched non-symptomatic joints to control the effect of systemic improvement, such as dilated blood vessels.

By using the agreed consensus PsADECTS score and iodine uptake, the treatment effects were calculated by Wilcoxon signed-rank test. The agreement of the improvement in PsADECTS score and iodine uptake was calculated with Spearman’s correlation coefficient.

Inter-reader reliability for PsADECTS was calculated with κ-values. κ-values less than 0.20 were considered to indicate poor agreement; values of 0.21–0.40 indicated fair agreement; values of 0.41–0.60 indicated moderate agreement; values of 0.61–0.80 indicated good agreement; and values of 0.81 and higher indicated excellent agreement. The 95% confidence interval for inter-reader agreement was calculated by using a bootstrap bias-correction method with 1000 bootstrap replications. Inter-reader reliability for iodine quantification was calculated with Wilcoxon signed rank test.

To evaluate changes of variables of clinical parameters between the baseline and the designated time point, the Wilcoxon signed rank test was used.

To calculate the correlation between the changes of imaging and clinical findings, the most symptomatic joint according to the iodine map, of each patient was selected to match the blood results. The correlation was calculated with Spearman’s correlation coefficient.

All the statistical data was performed with STATA version 14.2 (StataCorp). p value of less than 0.05 was considered significant.

## Results

A total of 26 consecutive patients who had symptomatic PsA of digital joints and completed two DECTs were included. The group included 17 men and 9 women, with a mean age of 50.6 years (range 22–89 years). The mean duration of PsA was 13.1 years (range 0–41 years), and the mean symptom duration was 26.9 months (range 1–192 months). The mean interval between the two DECTs was 7.0 months (range 4–12 months). The mean interval between the blood sample and DECT was 8.02 ± 16.29 days. All patients fulfilled the CASPAR criteria. There were no significant differences in the clinical characteristics between men and women (Table 2). Break down of the medications used and the number of patients were as follows; adalimumab: 9, infliximab: 8, secukinumab: 7, methotrexate: 1, infliximab and methotrexate: 1.

**Table 2:** Patient demographics and characteristics.

Characteristic	All patients	Men	Women	p
Number of patients	26	17	9	
Mean age (year)	50.6 (22–89)	54.22 (25–89)	45.17 (22–68)	0.89
Mean psoriasis duration (year)	13.13 (0–41)	12.33 (0.5–35)	14.13 (0–41)	0.87
Mean current joint symptom duration (month)	26.88(1–192)	40.3 (1–192)	24.8 (1–120)	0.64
Mean interval between the two DECTs (month)	7.02 (4–12)	7 (4–12)	6.35 (4–11)	0.97
Mean PASI score	3.78 (0–27.3)	2.85 (0–10.2)	5.54 (0–27.3)	0.67
Fulfilled CASPAR criteria	26	17	9	0.21
Mean C-reactive protein level (mg/dL)	1.11 (0.04–6.05)	0.98 (0.04–5.45)	1.34 (0.04–6.05)	0.5
Nail change	16	11	5	0.69

Note.- Numbers in parentheses are ranges. CASPAR = Classification Criteria for Psoriatic Arthritis, PASI = Psoriasis Area Severity Index. To convert milligrams per deciliter to nano moles per liter, multiply by 9.524. *P < 0.05 was considered statistically significant.

The mean and standard deviation of dose length product (DLP) and CT dose index (CTDIvol) of available data (n = 19) were 273 ± 19.31 mGycm and 10.73 ± 0.26 mGy, respectively.

DECT iodine map of 74 symptomatic and 74 non-symptomatic joints were analyzed. Significant improvements were seen in both PsADECTS score and iodine uptake after treatment (from 4.12 ± 2.29 to 1.01 ± 1.60, Wilcoxon signed-rank test: z = 7.38, and from 6.04 ± 4.43 mg to 1.77 ± 3.02 mg, Wilcoxon signed-rank test: z = 6.20, respectively. p < 0.005 for both z) (Table 3).

**Table 3:** Agreed consensus results.

	Pre-treatment	Post-treatment	Treatment effect	Wilcoxon signed-rank test
Total Score of PsADECTS	4.12 ± 2.29	1.01 ± 1.60	3.11 ± 1.79	z = 7.38 (p < 0.005)
Iodine uptake (mg)	6.04 ± 4.43	1.77 ± 3.02	4.28 ± 3.75	z = 6.20 (p < 0.005)

Data represent mean ± standard deviation (SD).

**Figure 3:**
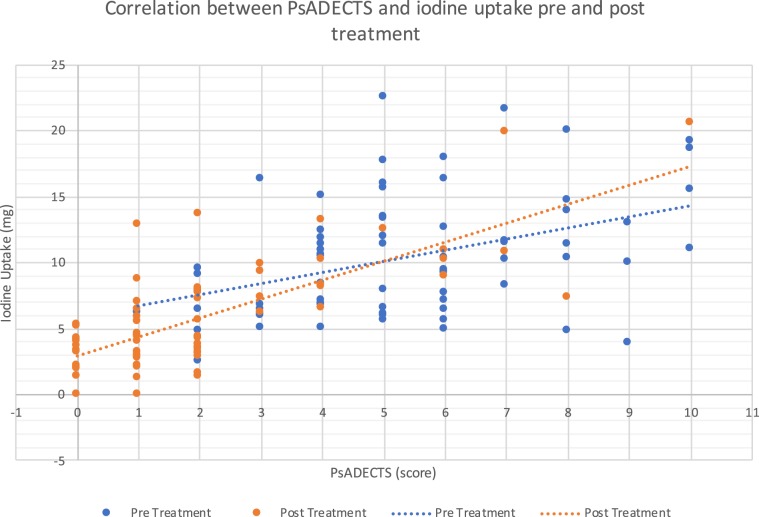
Correlation between PsADECTS and iodine uptake pre and post treatment. Decreasing value of both PsADECTS and iodine uptake after treatment are suggestive of overall improvement of digital PsA.

**Figure 4:**
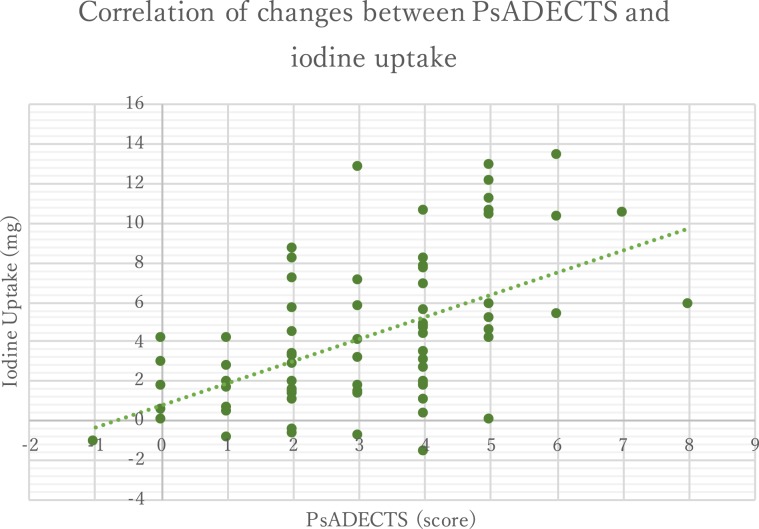
The treatment effect of PsADECTS score and iodine uptake correlation. PsADECTS score and iodine uptake showed a good correlation (Spearman’s ρ = 0.58 p < 0.005). Iodine uptake results showed greater changes compared to the relatively small changes in the PsADECTS score, which may indicate that iodine uptake is a more sensitive measure of the inflammatory activity of PsA.

PsADECTS and iodine uptake both decreased in value after treatment (Fig. 3). The treatment effects of PsADECTS score and iodine uptake showed a good correlation (Spearman’s ρ = 0.58 p < 0.005) (Fig. 4).

Inter-reader agreement of PsADECTS score for symptomatic joints pre and post treatment were moderate and good agreement, respectively (weighted κ = 0.57 [95% confidence interval {CI}: 0.49–0.65] for pre-treatment; weighted κ = 0.74 [95% confidence interval {CI}: 0.61–0.80] for post-treatment). Inter-reader agreement of iodine uptake for symptomatic joints for both pre and post treatment showed good correlation (Spearman’s ρ = 0.92 p = < 0.0001, Spearman’s ρ = 0.95 p = < 0.0001, respectively) (Table 4).

**Table 4:** PsADECTS and Iodine uptake of symptomatic and non-symptomatic joints, pre and post treatment.

		Pre-treatment		Inter-reader reliability	Post-treatment		Inter-reader reliability
		Reader 1	Reader 2		Reader 1	Reader 2	
Symptomatic joints	Total Score of PsADECTS	5.45 ± 2.29	5.18 ± 2.29	weighted κ (95% CI) = 0.57 (0.49–0.65)	2.03 ± 2.13	1.96 ± 2.08	weighted κ (95% CI) = 0.70 (0.61–0.80)
	Iodine uptake (mg)	9.71 ± 4.18	10.26 ± 4.49	Spearman’s ρ = 0.92 p < 0.0001	5.60 ± 3.72	5.71 ± 4.00	Spearman’s ρ = 0.95 p < 0.0001
Non-symptomatic joints	Total Score of PsADECTS	2.04 ± 1.88	1.05 ± 0.83	weighted κ (95% CI) = 0.36 (0.26–0.45)	1.48 ± 1.28	0.95 ± 0.95	weighted κ (95% CI) = 0.44 (0.32–0.57)
	Iodine uptake (mg)	4.24 ± 1.94	4.21 ± 2.04	Spearman’s ρ = 0.86 p < 0.0001	4.01 ± 2.06	3.95 ± 2.06	Spearman’s ρ = 0.92 p < 0.0001

Data represent mean ± standard deviation (SD).

Inter-reader agreement of PsADECTS score for non-symptomatic joints pre and post treatment were fair and moderate, respectively (weighted κ = 0.36 [95% confidence interval {CI}: 0.26–0.45] for pre-treatment; weighted κ = 0.44 [95% confidence interval {CI}: 0.32–0.57] for post-treatment). Inter-reader agreement of iodine uptake for non-symptomatic joints both pre and post treatment showed good correlation (Spearman’s ρ = 0.86 p = < 0.0001, Spearman’s ρ = 0.92 p = < 0.0001, respectively).

Compared with baseline values, clinical parameters, VAS, CRP, platelets, lymphocytes, PLR and NLR showed significant decreases after treatment (p < 0.005) (Table 5).

**Table 5:** Comparison of clinical variables between pre and post treatment.

	Pre-treatment		Post-treamtment		*p*
	Mean	SD	Mean	SD	
VAS	41.73	19.34	14.62	14.49	<0.001**
UA (mg/dL)	5.22	1.41	5.49	1.36	n.s.
CRP (mg/dL)	1.14	2.02	0.27	0.62	<0.05*
MMP-3 (ng/mL)	119.92	143.19	74.48	39.49	n.s.
WBC (x103/mL)	7.3	1.53	7.06	1.77	n.s.
Plt (x103/mL)	278.35	90.71	248.92	67.22	<0.05*
Neu (x103/mL)	4.67	1.56	4.09	1.28	<0.05*
Lym (x103/mL)	2	0.82	2.15	0.83	<0.05*
PLR	170.07	116.4	130.13	53.09	<0.001**
NLR	2.97	2.28	2.24	1.66	<0.05*

*P < 0.05 was considered statistically significant. VAS, Visual Analog Scale; UA, Uric acid; CRP, serum C-reactive protein; MMP-3, matrix metalloproteinase-3; WBC; white blood cell; Plt, Platelets; Neu, Neutrophils; Lym, Lymphocytes; PLR, platelet-lymphocyte ratio; NLR, neutrophil-lymphocyte ratio. CRP was not available in two cases.

Table 6 shows the correlations between clinical variables and imaging findings. For the pre-treatment set of results, PsADECTS score showed a significant correlation with pre-treatment CRP (Spearman’s ρ = 0.45 p < 0.05), whereas with iodine uptake showed correlations with pre-treated values of VAS and PLR (Spearman’s ρ = 0.54, Spearman’s ρ = 0.51, respectively. p < 0.05). For the post-treatment set of results, PsADECTS score did not show any significant correlation with the clinical data. When compared with post-treatment iodine uptake and clinical criteria, VAS showed a significant correlation (Spearman’s ρ = 0.43, p < 0.05) (Table 6).

**Table 6:** Correlation between clinical variables and imaging findings.

	Pre-treatment				Post-treatment				Values of treatment effect			
	PsADECTS score		Iodine uptake		PsADECTS score		Iodine uptake		PsADECTS score		Iodine uptake	
	Spearman’s ρ	p	Spearman’s ρ	p	Spearman’s ρ	p	Spearman’s ρ	p	Spearman’s ρ	p	Spearman’s ρ	p
VAS	0.33	n.s.	0.54	<0.05*	0.28	n.s.	0.43	<0.05*	0.05	n.s.	−0.1	n.s.
UA	0.01	n.s.	−0.14	n.s.	−0.26	n.s.	−0.27	n.s.	−0.1	n.s.	−0.04	n.s.
CRP	0.45	<0.05*	0.38	n.s.	0.02	n.s.	−0.04	n.s.	−0.14	n.s.	−0.16	n.s.
MMP-3	0.31	n.s.	0.3	n.s.	−0.12	n.s.	−0.15	n.s.	0.11	n.s.	0.1	n.s.
WBC	0.34	n.s.	0.24	n.s.	−0.17	n.s.	−0.23	n.s.	−0.52	<0.05*	−0.44	<0.05*
Plt	0.31	n.s.	0.41	n.s.	0.29	n.s.	0.17	n.s.	0.15	n.s.	0.13	n.s.
Neu	0.3	n.s.	0.24	n.s.	−0.04	n.s.	−0.11	n.s.	−0.31	n.s.	−0.36	n.s.
Lym	0.17	n.s.	−0.24	n.s.	0.13	n.s.	0.07	n.s.	−0.25	n.s.	−0.04	n.s.
PLR	0.15	n.s.	0.51	<0.05*	0.07	n.s.	0.03	n.s.	0.19	n.s.	0.27	n.s.
NLR	0.14	n.s.	0.34	n.s.	−0.11	n.s.	−0.12	n.s.	−0.24	n.s.	−0.19	n.s.

All values represent Spearman’s ρ. *p < 0.05 was considered statistically significant. VAS, Visual Analog Scale; UA, Uric acid; CRP, serum C-reactive protein; MMP-3, matrix metalloproteinase-3; WBC; white blood cell; Plt, Platelets; Neu, Neutrophils; Lym, Lymphocytes; PLR, platelet-lymphocyte ratio; NLR, neutrophil-lymphocyte ratio. CRP was not available in two cases.

## Discussion

This study showed a significant improvement in PsADECTS score and iodine uptake after the treatment of PsA with significant correlation in the two methods. Moreover, a good inter-reader reliability was obtained when DECT iodine map was evaluated quantitatively and the inter-reader reliability was relatively consistent between symptomatic and non-symptomatic joints. Therefore, DECT iodine map can be a useful and efficient tool in evaluating the treatment effect of PsA.

In PsA, inflammation occurs predominantly at the enthesis, a site where tendons and ligaments adhere to bone, which may lead to a secondary inflammation in the synovium of adjacent joints and tendon sheaths, eventually causing bone erosion and joint destruction^19,20^. With the rapid development of treatment options, including biologic therapies, it is preferable to treat enthesitis and synovitis before bone erosion and joint destructions occur^21,22^. Although PsADECTS is a useful tool in evaluating the severity of PsA, and is shown to be as useful or may be superior in assessment of inflammation of small joint as MRI^17^, there is a degree of subjective element in the scoring process and also the scoring of each joint is time consuming. Consequently, developing a tool to assess the severity and treatment response in a more objective and efficient manner, before irreversible structural changes occur to lessen its unsightly and disabling progression, is desirable.

PsADECTS and iodine uptake both decreased after treatment, suggestive of overall improvement of digital PsA (Fig. 3). The treatment effect of PsADECTS score and iodine uptake showed a good correlation (Spearman’s ρ = 0.58 p < 0.005), suggesting that DECT iodine map can assist the evaluation of treatment effect in PsA. However, a large spread in values were observed in both PsADECTS and iodine uptake (Figs. 3 and 4).

As this was a retrospective study, we were unable to standardize the methods of treatment, which might have affected the result and a prospective study would be more appropriate to investigate the effect of treatment on iodine uptake. Furthermore, the interval between the two DECTs differed for each patient, which might have also affected the result. EULAR stated that the decision on when to repeat US/MRI depends on the clinical circumstances^6^. Therefore, it would be difficult to set a particular interval to investigate all patients. However, a further study would be desirable to investigate how treatment duration affects iodine accumulation.

Furthermore, while scoring the images, the scores did not always relate to the strength of enhancement. Even when there was a florid enhancement around the joints suggestive of strong inflammation, if the enhanced structure did not fulfil the scoring criteria, the score may not be as high as expected, and vice versa. The scoring system is also a subjective assessment, and the score may differ among the assessors and their length in experience in interpreting musculoskeletal images. The quantification of iodine uptake is more objective with constantly high inter-reader reliability, and can be assessed in continuous variables, which may be a more sensitive and objective assessment in reflecting changes of inflammation which may not be sufficient enough to affect the scores. In this study, the inter-reader reliability was relatively constant in both symptomatic and non-symptomatic joint for both pre and post-treatment when iodine was quantitatively measured compared to when it was assessed with PsADECTS. Therefore, assessing the severity of inflammation with scoring system may involve a risk of under or overestimation of the inflammation depending on the assessor.

The spread of iodine uptake may also be partly attributed to the accuracy of material decomposition^23–26^. One of the reasons for the limited accuracy of quantification in dual source DECT could be due to the use of the image-based method instead of the raw data-based method. Image-based methods are inferior to raw data-based methods because the ability to perform an exact material separation that combines the polychromatic properties of both spectra is lost^23^. Amplified image noise is also an issue for image-based material decomposition^24^. Dual source DECT cannot process the raw data sets, because it does not allow measurements on geometrically identical lines with each spectrum, due to its detector arrangements. Therefore, information needs to be analyzed on image domain after image reconstruction. Material decomposition from inconsistent rays is an iterative method to indirectly perform raw data-based DECT even though different lines were measured for both spectra^23^. This method may reduce artifacts and improve mean density errors in material density images in the near future. Other methods of improving accuracy of material decomposition, such as the development of algorithms to reduce image noise, use of triple energy CT and photon counting detector are also investigated^24–26^.

In this study, one case showed worsening with both PsADECTS score and iodine uptake, and a few cases showed unchanged or improved PsADECTS score with worsening iodine uptake. This could be due to worsening subclinical inflammation of the non-symptomatic control joint affecting the results. When the PsADECTS score and the value of iodine uptake were subtracted from those of the control joint, although there was slight improvement in the symptomatic joint, inflammation of the control may mask the improvement. Subclinical joint inflammation has been mentioned in previous studies^27,28^, and a follow-up study to investigate the relapse rate in those patients would be of interest, which may reveal the importance of detecting subclinical joints at an early stage. In several cases, especially in asymptomatic joints, changes in iodine uptake were observed despite the absence of changes in the PsADECTS scores. These results may suggest that quantifying iodine uptake may provide a more detailed measure of inflammatory activity of PsA. A prospective study to evaluate the correlation between the imaging and clinical findings should be performed to investigate this.

Clinical variables also showed an overall improvement, excluding uric acid (UA), matrix metalloproteinase-3 (MMP-3) and white blood cell (WBC), which is consistent with the imaging findings. However, when the correlation between imaging findings and clinical findings were evaluated, the correlation was limited. VAS correlated only with iodine uptake in both pre and post treatment, which may suggest that quantification correlate more with clinical findings. This limited correlation could be due to the fact that, there was a time lag between when the DECT was performed and when the blood test was taken (8.02 ± 16.29 days). Furthermore, PsA is a systemic disease that affects many joints, including axial joints. A previous study showed that the presence of axial involvement was associated with higher disease activity, including CRP and VAS^29^. Hence, results of the blood samples may not completely reflect the state of joints in the hand and foot. Moreover, due to the retrospective nature of this study, post-treated PASI score was not performed in all patients. However, there is a study reporting the correlation of NLR and PLR with the PASI scores in PsA patients^18^. Due to the time lag between the time of imaging and the clinical findings, the correlation of the two was only weak, and we can speculate that the overall improvement in blood samples including NLR and PLR, and imaging findings represent the improvement of PsA post-treatment.

It is important to note that DECT holds several disadvantages. Firstly, DECT involves radiation exposure. Our cases were scanned with dual-source DECT, allowing the use of tube current modulation and tin filters with high-energy spectrum to reduce radiation dose. Several studies have shown that the radiation dose of dual-source DECT is maintained similar to the radiation dose when compared with the conventional single-energy multidetector CT^30–34^. Also, hands and feet are distant to radiosensitive organs, such as the lens or abdominal organs, and therefore they can be scanned by CT repeatedly at an appropriate interval with relative safety. Secondly, the accessibility of DECT is facility-dependent compared with US or MRI. Finally, the use of iodine contrast material requires careful assessment of risks, such as renal function and history of allergy. However, contrast medium is also required even with an MRI for accurate evaluation of inflammatory arthritis^35^.

There are several limitations in our study. First, limitation of our study was the lack of a histopathologic reference standard as it is ethically questionable to perform 2 biopsies pre and post treatment in PsA patients. However, clinical findings in the form of VAS, CRP, NLR and PLR showed significant improvement with treatment, which is consistent with the image findings. The second limitation is the small sample size, varied medications used for treatment and the time difference between obtaining images and clinical data, due to the retrospective nature of the study. To overcome this problem, we have investigated multiple joints from each patient to maximize evaluation site. For a more precise evaluation between the correlation of imaging and clinical findings, a prospective study should be conducted to reduce the time lag in between DECT and blood tests, with a larger sample size after standardizing medication used for treatment. The third limitation is the absence of correlation with MRI. However, a previous study has already evaluated the feasibility of iodine map in evaluation of PsA when compared with contrast enhanced MRI, which showed that the iodine map may be as useful as MRI or may be more superior in detection of inflammatory lesions in small joints^17^. Finally, although the iodine uptake quantification is a more objective measure of inflammation in PsA, there is a small component of subjective measure, particularly when determining the position of the ROI. Currently, iodine quantification can only be measured on 2 dimensional images, therefore the value of iodine uptake may not represent the degree of inflammation of the whole joint.

In conclusion, our study showed that the DECT iodine map is a valid tool for quantitative assessment of the therapeutic responses of PsA.

## Data Availability

The datasets generated during and/or analyzed during the current study are available from the corresponding author on reasonable request.
